# Relationships between Resisted Sprint Performance and Different Strength and Power Measures in Rugby Players

**DOI:** 10.3390/sports8030034

**Published:** 2020-03-14

**Authors:** Santiago Zabaloy, Jorge Carlos-Vivas, Tomás T. Freitas, Fernando Pareja-Blanco, Lucas Pereira, Irineu Loturco, Thomas Comyns, Javier Gálvez-González, Pedro E. Alcaraz

**Affiliations:** 1 Faculty of Physical Activity and Sports, Universidad de Flores, Pedernera 268, Buenos Aires, Argentina; 2 Faculty of Sports Sciences, Universidad Pablo de Olavide, Carretera Utrera km 1, 41013 Seville, Spain; fparbla@gmail.com (F.P.-B.); jgalgon@upo.es (J.G.-G.); 3 UCAM Research Center for High Performance Sport, Catholic University of Murcia, Campus de los Jerónimos, 30107 Guadalupe, Murcia, Spain; jorge.carlosvivas@gmail.com (J.C.-V.); tfreitas@ucam.edu (T.T.F.); 4 NAR—Nucleus of High Performance in Sport, São Paulo, SP 04753-070, Brazil; lucasa_pereira@outlook.com (L.P.); irineu.loturco@terra.com.br (I.L.); 5 Department of Human Movement Sciences, Federal University of São Paulo, Butanta, SP 11015-020, Brazil; 6 Physical Performance & Athletic Research Center, Universidad Pablo de Olavide, Carretera Utrera km 1, 41013 Seville, Spain; 7 Faculty of Life Sciences and Education, University of South Wales, Pontypridd, Wales CF37 1DL, UK; 8 Department of Physical Education and Sport Sciences, University of Limerick, V94 T9PX Limerick, Ireland; Tom.Comyns@ul.ie; 9 Faculty of Sport Sciences, UCAM, Catholic University of Murcia, Campus de los Jerónimos, 30107 Guadalupe, Murcia, Spain

**Keywords:** team sports, performance, testing, training, speed

## Abstract

This study aimed to investigate the relationship between a specific isometric-strength sprint test (SIST) and unresisted maximum velocity (Vmax), sprint times across different loading conditions, and the velocity loss (Vloss) loads required to achieve each intended Vloss condition during resisted sprint training (RST) in rugby players. Additionally, the investigation examined the relationship between strength in the back-squat one-repetition maximum (1RM-SQ) as well as isometric squat (ISQT), jumps, and sprint performance variables. Twenty (n = 20) male amateur rugby players performed, on two separate occasions, a structural multiple-joint assessment of jumps, strength, and sprint performance. Interestingly, SIST revealed moderate correlations (r = 0.453 to 0.681; *p* < 0.05) between 1RM-SQ and ISQT. The SIST_rel_ (relative to body mass), but not SIST, used in the present study showed moderate correlations (r = 0.508 to 0.675; *p* < 0.05) with the loads needed to reach 10%, 30%, and 50% of Vloss during RST. The SIST_rel_ that measures resultant force application in a more sprint-related position explains much of the individual response of each athlete during sprinting towing a sled and can also be used to prescribe and quantify loads in the RST in a more objective and individual manner.

## 1. Introduction

Rugby union is a physically intense intermittent sport in which high-force collisions are common in match-play [1], thus requiring players to possess high levels of strength, power, and speed, regardless of playing position [2]. In addition, the game is characterized by multiple high-intensity actions (e.g., accelerations and maximum sprints) interspersed by low-intensity efforts [3,4].

In the literature, resisted sprint training (RST) is gaining attention and relevance for training in sports, aiming to improve speed and power. On the other hand, there is a growing amount of research in RST using sleds, although adequate load prescription requires further attention [5]. Harrison & Bourke [6] used 13% of body mass (BM) and 30 m sprints to analyse the effects of RST on speed and lower limbs strength in rugby players, and concluded that RST protocols may be useful to improve acceleration ability. According to Behrens & Simonson [7], the use of an appropriate load would be an important requirement to produce positive adaptations without drastically altering sprint mechanics. Traditionally, absolute loads (kg) or percentage of BM have been used to quantify the load in RST, but these methods do not account for the variability between subjects, the velocity loss, and the performance decrement that it targets with respect to the unloaded sprint [7,8,9]. Therefore, the percentage of Vloss with respect to the unloaded sprint has been proposed as a more appropriate method to normalize the stimulus applied to each athlete [10,11,12]. In addition, an athlete’s sprint time in a sled-towing exercise is affected by the coefficient of friction of the running surface and by the sled weight [13], making load prescription a more complex issue for practitioners.

In sport and everyday activities, one of the most important attributes of skeletal muscle is the ability to generate power, a product of strength and speed of movement [14]. Research has shown a strong relationship between strength, 20 m sprinting, and jumping performance in athletes and field sports athletes [15,16]. Another study [17] reported that a strong correlation exists between half-squat maximum strength, 30 m sprint performance, and jump height in soccer players. Moreover, a recent study reported that isometric mid-thigh pull (IMTP) variables are significantly associated with strength, agility, and sprint in rugby union players [2,18]. In contrast, the authors of [19] reported non-significant correlations between the squat jump (jump height) and 20 m sprint times in male and female rugby players. Nevertheless, it is clear that force production is an integral component to maximal sprinting velocity [20,21]. In rugby players, short sprint performance (5 m) was most strongly related to the proportion of maximal force achieved in the initial phase of isometric squats, while jump height was most strongly related to absolute force in the later phase of the explosive-isometric squats [22].

Strength expressed in both absolute terms and relative to BM has been considered as a critical factor for successful performances in Rugby union [23]. Authors also suggest that heavier athletes could focus on improving their power to BM ratio to positively influence jumping and sprinting abilities [24]. Increasing lower limbs’ strength, power, and muscle mass through appropriate resistance training while maintaining or improving speed may be useful to provide players with the underlying performance characteristics to play at an elite level in rugby [25].

However, recent research [18,22] studied explosive-isometric force production during traditional isometric tests (i.e., IMTP and squat), which are in the vertical plane, although sprinting (especially acceleration) is predominantly horizontal in direction. This lack of specificity could be addressed through the design and use of a specific isometric-strength sprint test (SIST). Therefore, to further explore the relationship between dynamic and isometric strength capabilities and sprint performance, it would be relevant to design a specific test to measure resultant force application in a more sprint-related position. As the RST method allows more horizontal force application [8], an isometric strength test performed in a specific position that allows the measurement of horizontally-oriented force could potentially explain, to an important extent, the individual response of each athlete to different RST loading conditions.

In light of the above, it is plausible to infer that an especially designed SIST would correlate not only with sprint performance, but also with the loads needed to achieve 10%, 30%, and 50% Vloss conditions. Therefore, the purpose of this paper was twofold: (1) to investigate the relationship between SIST and unresisted maximum velocity (Vmax), sprint times across different loading conditions, and the Vloss loads used during RST in rugby players; (2) to analyse the relationships between a structural multiple-joint assessment of strength (isometric and dynamic), jumps, Vmax, RST sprint times across different loading conditions, and the Vloss loads used during RST. Given the known relationship between jumping, sprinting velocity, and strength in rugby players, we hypothesized that lower body strength (dynamic and isometric) would correlate to sprint performance.

## 2. Materials and Methods

### 2.1. Experimental Approach to the Problem 

A descriptive-correlational cross-sectional study was performed. Assessments were conducted on two non-consecutive days, with 72 h of rest to avoid possible interferences caused by fatigue. On the first day, 30 m sprint tests were performed with different loads (i.e., 0%, 20%, 40%, 60%, and 80% BM, randomly applied) in order to obtain an individual linear regression equation able to specify the loads required to achieve each intended Vloss condition (10%, 30%, and 50% of Vloss). On day two, countermovement jump (CMJ), squat jump (SJ), and dynamic (i.e., 1RM Squat) and isometric (i.e., squat and SIST) strength assessments were conducted.

### 2.2. Participants

Twenty male, healthy, and active amateur rugby players (age: 22.5 ± 5.3 years; height: 1.80 ± 0.05 m; body mass: 80.2 ± 15.2 kg) with more than five years of competitive regional-level experience were involved in the study. The inclusion criteria were as follows: healthy and trained rugby players; no injuries or medical conditions in the last six months; and at least one year of previous experience in resistance and RST. During the research period, participants were required not to engage in any other type of strenuous physical activity, exercise training, or sports competition. All participants rested the day before testing and were asked to attend testing in a fed and hydrated state, similar to their normal practices before training. The study met the ethical standards and was approved by an Institutional Research Ethics Committee (CE031917) and conformed to the recommendations of the Declaration of Helsinki. After being informed of the purpose and experimental procedures, participants signed an informed consent form prior to participation.

### 2.3. Procedures

Testing on day one was conducted during the evening on an outdoor natural turf rugby pitch in dry weather conditions. Participants used their own training clothes and rugby cleats. Before the assessments, all participants performed a specific standardized warm-up, consisting of 10 min running at low-moderate intensity; 5 min active dynamic stretching; ~4 submaximal CMJs; and ~4 submaximal sprints over 10, 20, and 30 m, ensuring an interval of 90 s rest between each one. After the warm-up, also ensuring a 3 min recovery period, participants performed the unloaded sprints to avoid a possible potentiation effect on the following trials and, subsequently, the other loading conditions in a randomized order. Participants completed the tests at the same time of day. On day two, for jump and strength assessments, participants visited the indoor high-performance center. The order of testing was as follows: firstly the CMJ and SJ assessment, followed by isometric strength testing, which consisted of SIST and isometric squat test (ISQT). Finally, 1RM estimation in the squat exercise (1RM-SQ) was performed. Each testing stage was separated by an 8 min recovery period.

#### 2.3.1. Sprint Test for Individual Regression Equation Computation

Two 30 m sprints for each load condition with ~4 min of rest between trials were performed. Participants started on a two-point stance position, with the front foot 1 m behind a line away from the first timing gate. Photoelectric cells (Microgate, Bolzano, Italy) were placed using a tripod at 1 m height at the starting line and at 5, 10, 20, 25, and 30 m. In addition, a radar gun (Stalker ATS II, Applied Concepts, Richardson, TX, USA) was used to measure instantaneous velocity using a sampling frequency of 47 Hz. The radar was placed 5 m behind the starting line using a tripod at 1 m height. A sled towing device (Power systems, Power sled, weight: 13.4 kg) fixed to the athlete with a belt close to the centre of mass (CoM) and a 3.5 m long strap attached to the sled was used. Participants were encouraged to run 30 m as fast as possible. The best time in 30 m in each loading condition was used for the subsequent analysis and named as follows: T_30_ (unresisted), T_30_-20BM (20% BM), T_30_-40BM (40% BM), T_30_-60BM (60% BM), and T_30_-80BM (80% BM). The maximum velocity reached in the unloaded condition and the loads needed to reach 10% Vloss (L10%), 30% Vloss (L30%), and 50% Vloss (L50%) were also used in the analysis. Each participant’s load condition was obtained according to their individual regression equation computation.

#### 2.3.2. Jump Tests

Participants performed two vertical jump tests (SJ and CMJ) assessed on a force plate (Kistler 9286BA, Winterthur, Switzerland) sampling at 350 Hz. The variables obtained during these tests were as follows: maximum jump height (JH) and peak power relative to BM (PP_rel_). The warm-up protocol consisted of 5 min low-intensity jogging, joint mobility, and dynamic stretching; followed by 10 min of non-fatiguing activation and mobilization exercises, including bodyweight lunges and squats; and, finally, two sets of four submaximal repetitions of SJ and CMJ. Participants then performed three CMJ and three SJ with hands on the hips, separated by 15 s and 4 min of recovery between tasks [26]. The highest JH value in each task was kept for the subsequent analysis.

#### 2.3.3. Specific Isometric Strength Test

**Figure 1:**
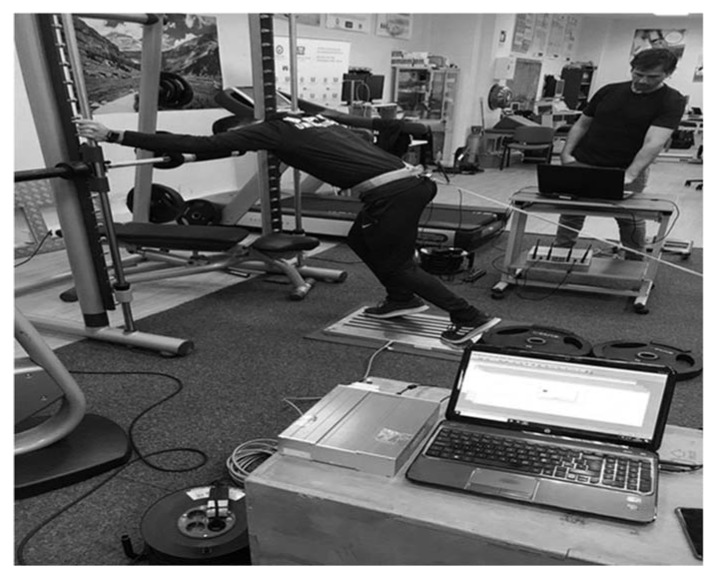
Model of the pilot tests performed for the specific isometric strength test.

**Figure 2:**
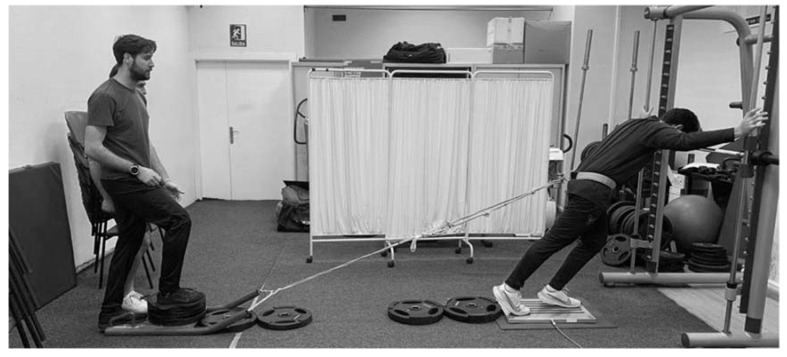
Model of the pilot tests performed for the specific isometric strength test.

SIST was performed using a sled towing device (Power systems, Power sled, weight: 13.4 kg) fixed to the athlete with a belt close to the CoM and a 3.5 m long strap attached to the sled. Additionally, the sled was loaded with ~300 kg to prevent it from moving forward. A surface of non-slip material was adhered to the force plates to prevent participants sliding. Resultant mean force (N) was reported as the mean maximum force generated during the three second trial minus the subject’s body weight. Mean force in the SIST test was also reported relative to body mass (SIST_rel_). Participants started each repetition from a standing position similar to the starting position of the sprint tests on a force plate (Kistler 9286BA, Winterthur, Switzerland) sampling at 350 Hz (Software, BioWare V5.4.2.0, Kistler Ibérica S.L, Barcelona, Spain). The preferred foot positioned in front with comfortable trunk, thigh, and leg angles. The arms were placed holding a vertical bar (pushing or pulling was not allowed) of the Smith Machine to avoid unnecessary movements during each repetition. Participants were verbally encouraged to perform each repetition by applying maximum force during three seconds, avoiding any movement in the joints at all times. Two repetitions were performed, separated by ~5 min of recovery between each trial. The best trial was used for the subsequent analysis. Figure 1 and Figure 2 shows the pilot tests carried out prior to the data collection.

#### 2.3.4. Isometric Squat Strength Test

ISQT was performed on a force plate (Kistler 9286BA, Winterthur, Switzerland) sampling at 350 Hz (Software, BioWare V5.4.2.0, Kistler Ibérica S.L, Barcelona, Spain). Participants were positions on a Smith Machine, holding a maximal isometric contraction for at least three seconds and a knee angle approximately at 90º [18]. Mean force (N) was reported as the mean maximum force generated during the three second trial minus the subject’s body weight. Mean force in the ISQT assessment was also reported relative to body mass (ISQT_rel_). Participants were instructed to push as hard and as fast as they can to ensure that maximum force was achieved. Participants completed two trials and were instructed to get ready and were then given a countdown of “3, 2, 1, push!”. Verbal encouragement was provided during the trials. Two repetitions were performed, separated by ~5 min of recovery between each trial. The best trial was used for the subsequent analysis.

#### 2.3.5. Squat 1RM Estimation

After a 10 min rest, participants completed an incremental load test for 1RM estimation in squat exercise (1RM-SQ), using a validated linear position transducer (Chronojump, Boscosystem, Barcelona, Spain) [27]. The 1RM estimation was based on the equation previously reported [28]. Owing to the very close relationship (R² = 0.95) between %1RM and mean propulsive velocity (MPV) in the full squat exercise, the 1RM was estimated from the MPV attained against the heaviest load of the test, as follows: %1RM = −5.961 · MPV2 – 50.71 · MPV + 117.0 (SEE = 4.0% 1RM) [29]. Assessment was performed on a Smith Machine with the barbell resting on the upper part of the back and started with a deep flexion of the lower limbs until the thigh surpassed the horizontal with respect to the ground. Participants were instructed to perform concentric actions at maximal velocity and were not allowed to jump or take the bar off the shoulders. The initial load in the squat was set at 40 kg for all participants and gradually increased by 5–10 kg. Three repetitions were executed with each load with an interval of 3 min rest between each set. When participants reached an average mean propulsive velocity close to 0.5 m·s^−1^, the test ended and the 1RM was estimated. Once 1RM-SQ was estimated, relative to BM strength in squat (1RM-SQ_rel_) was also used in the analysis.

### 2.4. Statistical Analysis

Standard statistical methods were used for the calculation of means and standard deviations (SD). Shapiro–Wilk test was used to analyse if the values were normally distributed. In the case of non-normality, Spearman correlations were used for these variables. Intraclass correlation coefficient (ICC) and the coefficient of variation (CV) with a confidence interval of the ICC at 95% were calculated to provide the reliability of all measurements. Acceptable reliability was determined at an ICC ≥ 0.8 and a CV ≤ 10% [29]. Relationships between jump, strength, and sprint variables were determined by Pearson product-moment correlation. The r values were interpreted as weak (≤0.39), moderate (≥0.40–0.69), or strong (≥ 0.70) [30]. Significance level was set at *p* ≤ 0.05. All statistical analyses were performed using SPSS software (version 21.0; SPSS, IBM Corp., Armonk, NY, USA).

## 3. Results

The results of the reliability analysis showed acceptable reliability for CMJ and SJ height (ICC: 0.949–0.988 and CV: 2.84%–6.10%), ISQT (ICC: 0.941 and CV: 9.64%), SIST (ICC: 0.953 and CV: 7.07%). Regarding reliability for sprint times, the results showed acceptable reliability for T5 (unloaded, ICC: 0.933 and CV: 1.81%) and T30 (unloaded, ICC: 0.933 and CV: 1.77%). Regarding normality test, the results showed T_30_-40BM, T_30_-80BM, 1RM-SQ, ISQT_rel_, SIST, and SIST_rel_ were not normally distributed (*p* < 0.05). All other measures in the present study were deemed normal (*p* > 0.05). Descriptive results are presented in Table 1.

Regarding the correlations between sprint times and loads and the variables obtained after the assessments (Table 2), the results showed moderate to strong correlations between jump height in CMJ and SJ and 1RM-SQ_rel_ (only CMJ), unresisted and RST sprint times, and Vmax (0.510, *p* < 0.05 to 0.905, *p* < 0.001). Jump height in SJ showed moderate correlations between L10% and L30%. Regarding strength variables, 1RM-SQ moderate to strong correlations were revealed between jump height in SJ and 1RM-SQ_rel_, ISQT, and ISQT_rel_ (0.501, *p* < 0.05 to 0.761, *p* < 0.001). No correlations were found between strength variables and RST loads, and only L10% demonstrated a statistically significant and moderate correlation with ISQT. In addition, all three loads were correlated with each other (0.565, *p* = 0.009 to 0.982, *p* < 0.001). Regarding unresisted and RST sprint times, L10% showed moderate to strong correlations between Vmax, T_30_, and T_30_ under all loading conditions (0.482, *p* < 0.05 and 0.724, *p* < 0.001).

**Figure 3:**
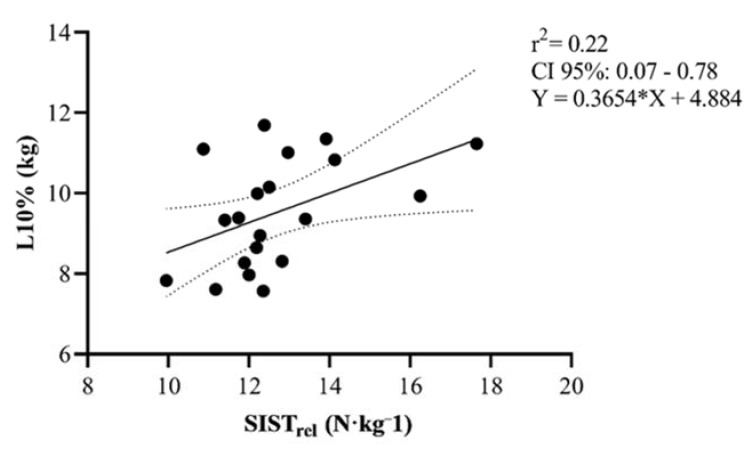
Scatter plot correlative findings between specific isometric-strength test relative to body mass (BM) (SIST_rel_) (N·kg^−1^) and the load needed to reach 10% Vloss during resisted sprinting among male rugby players. Dashed line demonstrates 95% confidence interval (CI).

**Figure 4:**
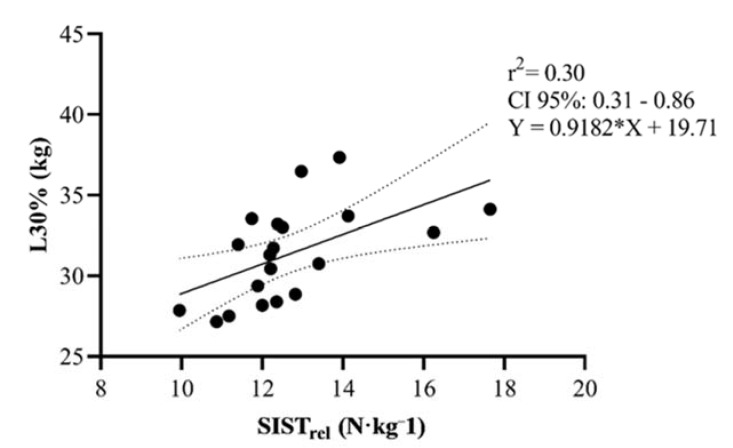
Scatter plot correlative findings between SIST_rel_ (N·kg^−1^) and the load needed to reach 30% Vloss during resisted sprinting among male rugby players. Dashed line demonstrates 95% CI.

**Figure 5:**
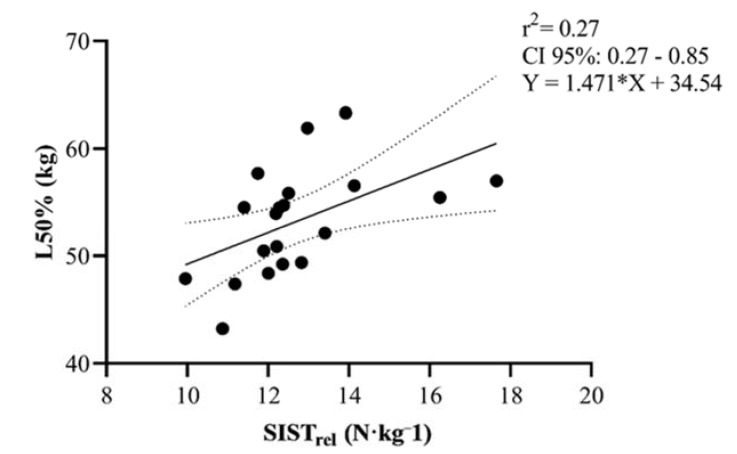
Scatter plot correlative findings between SIST_rel_ (N·kg^−1^) and the load needed to reach 50% Vloss during resisted sprinting among male rugby players. Dashed line demonstrates 95% CI.

Correlations between the SIST and SIST_rel_ and the remaining variables are presented separately in Table 3. Interestingly, SIST revealed moderate correlations (*p* < 0.001) between 1RM-SQ and ISQT. The SIST_rel_, but not SIST, used in the present study showed moderate correlations with the loads needed to reach 10%, 30%, and 50% of Vloss during RST *(*Figure 3, Figure 4 and Figure 5).

## 4. Discussion

The aims of the present study were to explore the relationships between SIST and unresisted maximum velocity (Vmax), sprint times across different loading conditions, and the Vloss loads used during RST in rugby players. In line with our hypothesis, this study showed that the SIST_rel_ designed for the present study is significantly correlated to the loads needed to reach 10%, 30%, and 50% of Vloss during RST. The present findings reveal that SIST_rel_ is a reliable test, and it is able to specifically measure a more horizontally-oriented force application, explaining much of the individual response of each athlete during RST. The SIST_rel_ can also be used to prescribe and quantify loads in RST, objectively and individually. 

The loads needed to reach 10% and 30% Vloss during RST demonstrated moderate correlations to SJ height. Overall, these correlations decreased as the loads increased. Moreover, moderate to strong correlations were found between Vmax, SJ, and CMJ (jump height), although Vmax was not associated to SIST or SIST_rel_. In addition, important correlations were found between jump height in both jump tasks and unresisted and RST times across all loading conditions (relative to BM).

Applying force in the horizontal direction may be beneficial for improving short sprint performance in professional rugby league players [31]. In the present study, important correlations were found between the loads needed to reach 10%, 30%, and 50% of Vloss, sprint, and jump variables. Our results indicate that Vmax showed moderate to strong correlations with JH in both jumps, which is in line with those found in elite sprinters [26], track and field athletes [32], professional rugby league players [33], and rugby union players [34]. These findings are supported not only by the fact that jump height was correlated to Vmax, but also because unresisted and RST times (i.e., T_30-_20BM, T_30-_40BM, T_30-_60BM, T_30-_80BM) were strongly correlated to both jumping tasks and Vmax. In contrast, a recent study in elite rugby players [19] reported no significant correlations between sprint times and jump height. The most noticeable difference between sprinting and jumping is that the former requires the resultant force and power to be more horizontally directed, while the latter requires a vertical orientation of the abovementioned variables [35]. Nevertheless, in the later phases of sprint, where the athletes are running close to or at Vmax, vertically oriented tasks (i.e., jumping) seem to be closely associated [32]. These findings have useful implications for practitioners in the way that JH and Vmax could be used not only to identify talent and monitor training, but also as resistance training strategies, where the aim should be placed on maximizing jumping ability and, hence, sprint performance.

Regarding RST loads, the present findings demonstrated that JHs in SJ, but not CMJ, were moderately correlated to L10% and L30%, but not to L50%, and this association decreased as a function of load increment. Previous research [36] reported that the increase in loads involves a decrease in sprint performance over 20 m when athletes sprinted under different conditions (unresisted and towing sleds with loads between 5% and 30% BM). The present findings suggest that, when sprinting with heavier loads, the variables that correlate with performance are different than when sprinting under unloaded conditions. This confirms that heavy sled training is quite different than unloaded sprint. In addition, JH during jumping is the variable that better explains much of the individual response of each athlete during unloaded and loaded sprinting with regards to times and Vmax. These results suggest that faster rugby players in the present study may possibly achieve greater CMJ and SJ heights.

Regarding the relationship between strength measures during 1RM-SQ and ISQT; Vmax; and the loads needed to reach 10%, 30%, and 50% of Vloss, the present findings showed that both strength tests are moderately correlated to each other, although no significant correlations were found with sprint variables. In fact, only 1RM-SQ_rel_ was shown to be moderately correlated with Vmax. In contrast to these results, a recent study in rugby players showed that measures of strength and power can be used to predict short sprint performance [37]. This suggests that traditional strength measures may be used to explain unloaded sprint performance, but not Vmax or the loads needed to reach a percentage of Vloss during RST. Additionally, the moderate correlations found between sprint and strength measures could be, in part, because of the homogenous sample, when considering that participants were all amateur rugby players.

To the authors’ knowledge, this is the first study to design a body-position specific test and investigate how it relates to athletes performance during unloaded and RST. An important finding in the present study is that both isometric tests (ISQT, SIST, and SIST_rel_) are moderately correlated to each other, and most importantly, SIST_rel_ showed moderate correlations to L10%, L30%, and L50%, but not to Vmax. This could be explained by the fact that isometric strength measures only correlate with sprint performance during the acceleration phase [18], but not to the later phases of the sprint. This information could help coaches to identify appropriate tests to monitor training effects, prescribing loads for RST, and to better assess an athlete’s specific strength–speed profile, which would help in future program design. Moreover, moderate to strong correlations were found between all loads, while L10% was the only load correlated to Vmax. As mentioned above, limiting factors of unloaded sprint performance seem to be quite different than those that determine heavy sled sprint performance.

There are some potential limitations to the current study, as future research should focus on test validation regarding SIST. The authors also highlight the fact that there was no familiarisation period previous to data collection and that no standardized angle position was used in SIST; however, all players were instructed to position themselves in a comfortable and adequate position, similar to that adopted during sprint starts. Along these lines, further studies with a larger sample of players and higher level population would be useful to investigate the potential effects of this novel test on the findings of the current study. The authors acknowledge that this study is the first step towards enhancing our understanding of the relationship between strength and sprint measures and the loads needed to reach a percentage of velocity loss during RST.

This study shows the relevance of performance in jumping tasks such as SJ, CMJ, and lower-body dynamic and isometric strength to performance in unloaded sprints (Vmax); RST sprint times; and the loads needed to reach 10%, 30%, and 50% of Vloss. Coaches and practitioners could better understand the training variables affecting sprint performance by including the SIST_rel_ in the assessment of strength-related capabilities. Indeed, as many factors influence the muscle’s ability to generate power, training for muscular power requires special care in developing the proper exercise prescription [14]. For coaches and practitioners involved in rugby, this test may be used to determine the athletes’ ability to tolerate RST loads during sprint-specific training and to design training sessions for developing rugby players’ specific lower body strength and power, which in turn could positively enhance sprint speed performance. It is recommended that future work should focus on replicating the specific isometric test and also consider that participants are set in the correct position (body position similar to a sprint start) and to avoid at all times any movement in the joints.

## 5. Conclusions

The SIST_rel_ that measures resultant force application in a more sprint-related position explains much of the individual response of each athlete during sprinting towing a sled and can also be used to prescribe and quantify loads in the RST in a more objective and individual manner. Moreover, moderate to strong correlations were found between Vmax, SJ, and CMJ (height), although Vmax was not associated to SIST or SIST_rel_.

## Figures and Tables

**Table 1:** Descriptive statistics for jumps, strength, and sprint assessments.

**CMJ** (cm)	**SJ** (cm)	**CMJ PP_rel_** (W·kg^−1^)	**SJ PP_rel_** (W·kg^−1^)
33.36 ± 6.28	30.09 ± 5.16	51.48 ± 6.67	49.65 ± 6.64
**1RM-SQ **(kg)	**1RM-SQ**_**rel**_ (kg·kg^−1^)	**ISQT**_**rel**_ (N·kg^−1^)	**ISQT** (N)
113.90 ± 23.73	1.41 ± 0.19	20.38 ± 4.68	1650.49 ± 521.97
**SIST**_**rel**_ (N·kg^−1^)	**SIST** (N)	**T**_**30**_ (s)	**T**_**30**_**-20BM** (s)
12.70 ± 1.76	1017.93 ± 246.66	4.32 ± 0.24	5.32 ± 0.42
**T**_**30**_**-40BM **(s)	**T**_**30**_**-60BM** (s)	**T**_**30**_**-80BM** (s)	**L10%** (kg)
6.45 ± 0.67	7.78 ± 1.00	10.30 ± 1.52	9.53 ± 1.36
**L30% **(kg)	**L50%** (kg)	**VMax** (m·s^−1^)	-
31.38 ± 2.93	53.23 ± 4.99	8.19 ± 0.55	-

CMJ: countermovement jump; SJ: squat jump; PP_rel_: peak power relative to body mass (BM); 1RM-SQ: 1RM estimated in the squat exercise; 1RM-SQ_rel_: 1RM relative to BM; ISQT_rel_: isometric squat test relative to body mass; ISQT: isometric squat test; SIST_rel_: specific isometric-strength test relative to BM; SIST: specific isometric-strength test; T_30_: unresisted 30 m sprint times; T_30_-20BM: 30 m sprint times during sled towing with 20% BM; T_30_-40BM: 30 m sprint times during sled towing with 40% BM; T_30_-60BM: 30 m sprint times during sled towing with 60% BM; T_30_-80BM: 30 m sprint times during sled towing with 80% BM; L10%: load needed to reach a 10% velocity loss; L30%: load needed to reach a 30% velocity loss; L50%: load needed to reach a 50% velocity loss; Vmax: maximum velocity reached in the unloaded condition.

**Table 2:** Correlation matrix between jumps, strength, and sprint variables obtained after the assessments in male rugby players.

Variables	Correlations													
	Jumps			Strength		RST Loads				Unresisted and RST Times				
	*CMJ*	*SJ*	*1RM-SQ*	*1RM-SQ_rel_*	*ISQT_rel_*	*ISQT*	*L10%*	*L30%*	*L50%*	*VMAX*	*T_30_*	T_30_-20BM	T_30_-40BM	T_30_-60BM
*SJ*	0.905 **	-	-	-	-	-	-	-	-	-	-	-	-	-
*1RM-SQ*	−0.186	−0.262	-	-	-	-	-	-	-	-	-	-	-	-
*1RM-SQ_rel_*	0.510 *	0.345	0.501 *	-	-	-	-	-	-	-	-	-	-	-
*ISQT_rel_*	0.198	−0.014	0.605 **	0.633 **	-	-	-	-	-	-	-	-	-	-
*ISQT*	0.132	−0.116	0.761 **	0.423	0.868 **	-	-	-	-	-	-	-	-	-
*L10%*	0.362	0.566 **	−0.369	−0.043	−0.444	−0.553 *	-	-	-	-	-	-	-	-
*L30%*	0.370	0.446 *	−0.146	0.073	−0.143	−0.302	0.712 **	-	-	-	-	-	-	-
*L50%*	0.336	0.370	−0.159	0.097	−0.128	−0.204	0.564 **	0.981 **	-	-	-	-	-	-
*VMAX*	0.747 **	0.795 **	−0.170	0.447 *	−0.014	−0.209	0.559 *	0.324	0.228	-	-	-	-	-
*T_30_*	−0.734 **	−0.787 **	0.084	−0.534 *	0.059	0.168	−0.560 *	−0.342	−0.249	−0.977 **	-	-	-	-
*T_30_-20BM*	−0.671 **	−0.744 **	0.090	−0.379	0.053	0.307	−0.724 **	−0.414	−0.288	−0.944 **	0.933 **	-	-	-
*T_30_-40BM*	−0.688 **	-0.645 **	0.183	−0.362	0.036	0.241	−0.638 **	−0.399	−0.409	−0.709 **	0.691 **	0.776 **	-	-
*T_30_−60BM*	−0.655 **	−0.728 **	0.210	−0.336	−0.059	0.279	−0.672 **	−0.633 **	−0.561 *	−0.839 **	0.800 **	0.787 **	0.695 **	-
*T_30_-80BM*	−0.833 **	−0.689 **	0.153	−0.340	−0.028	0.126	−0.482 *	−0.485 *	−0.505 *	−0.761 **	0.680 **	0.815 **	0.675 **	0.638 **

* Correlation is significant at the 0.05 level. ** Correlation is significant at the 0.01 level. BM: body mass; CMJ: countermovement jump; SJ: squat jump; 1RM-SQ: 1 RM estimated in the squat exercise; 1RM-SQ_rel_: 1RM relative to BM; ISQT_rel_: isometric squat test relative to body mass; ISQT: isometric squat test; L10%: load needed to reach a 10% velocity loss; L30%: load needed to reach a 30% velocity loss; L50%: load needed to reach a 50% velocity loss; Vmax: maximum velocity reached in the unloaded condition. T_30_: unresisted 30 m sprint times; T_30_-20BM: 30 m sprint times during sled towing with 20% BM; T_30_-40BM: 30 m sprint times during sled towing with 40% BM; T_30_-60BM: 30 m sprint times during sled towing with 60% BM; T_30_-80BM: 30 m sprint times during sled towing with 80% BM.

**Table 3:** Correlations between the SIST and SIST_rel_, jumps, strength, and sprint variables obtained after the assessments.

*Variables*	SIST_rel_			SIST		
	Correlations	*p*-Value	Description	Correlations	*p*-Value	Description
CMJ (cm)	0.211	0.373	weak	−0.328	0.158	weak
SJ (cm)	0.284	0.225	weak	−0.299	0.200	weak
CMJ-PP_rel_ (W·kg^−1^)	0.290	0.214	weak	−0.221	0.349	weak
SJ-PP_rel_ (W·kg^−1^)	0.293	0.210	weak	−0.281	0.231	weak
1RM-SQ (kg)	0.210	0.930	weak	0.674	<0.001 **	moderate
1RM-SQ_rel_ (kg·kg^−1^)	−0.039	0.870	weak	−0.023	0.925	weak
ISQT_rel_ (N·kg^−1^)	−0.071	0.767	weak	0.349	0.122	weak
ISQT (N)	−0.027	0.910	weak	0.681	<0.001 **	moderate
SIST_rel_ (N·kg^−1^)	-	-	-	0.453	0.045 *	moderate
SIST (N)	0.453	0.045 *	moderate	-	-	-
L10% (kg)	0.508	0.022 *	moderate	−0.302	0.195	weak
L30% (kg)	0.675	<0.001 **	moderate	−0.008	0.975	weak
L50% (kg)	0.645	0.002 **	moderate	−0.021	0.930	weak
VMax (m·s^−1^)	0.049	0.838	weak	−0.315	0.176	weak
*T*_*30*_ * (s)*	−0.120	0.613	weak	0.269	0.252	weak
*T*_*30*_ *-20%BM (s)*	0.014	0.952	weak	0.358	0.121	weak
*T*_*30*_ *-40%BM (s)*	−0.046	0.848	weak	0.453	0.045 *	moderate
*T*_*30*_ *-60%BM (s)*	−0.310	0.184	weak	0.239	0.310	weak
*T*_*30*_ *-80%BM (s)*	−0.037	0.877	weak	0.377	0.101	weak

* Correlation is significant at the 0.05 level; ** correlation is significant at the 0.01 level. BM: body mass; CMJ: countermovement jump; SJ: squat jump; PP_rel_: peak power relative to BM; 1RM-SQ: 1 RM estimated in the squat exercise; 1RM-SQ_rel_: 1RM relative to BM; ISQT_rel_: isometric squat test relative to body mass; ISQT: isometric squat test; SIST_rel_: specific isometric strength test relative to BM; SIST: specific isometric strength test; L10%: load needed to reach a 10% velocity loss; L30%: load needed to reach a 30% velocity loss; L50%: load needed to reach a 50% velocity loss; Vmax: maximum velocity reached in the unloaded condition. T_30_: unresisted 30 m sprint times; T_30_-20BM: 30 m sprint times during sled towing with 20% BM; T_30_-40BM: 30 m sprint times during sled towing with 40% BM; T_30_-60BM: 30 m sprint times during sled towing with 60% BM; T_30_-80BM: 30 m sprint times during sled towing with 80% BM.
